# Peculiarities and Applications of Stochastic Processes with Fractal Properties

**DOI:** 10.3390/s21175960

**Published:** 2021-09-05

**Authors:** Oleg Semenovich Amosov, Svetlana Gennadievna Amosova

**Affiliations:** 1 Laboratory of Intellectual Control Systems and Modeling, V.A. Trapeznikov Institute of Control Sciences of Russian Academy of Sciences, 65 Profsoyuznaya Street, 117997 Moscow, Russia; 2 Laboratory of Cyber-Physical Systems, V.A. Trapeznikov Institute of Control Sciences of Russian Academy of Sciences, 65 Profsoyuznaya Street, 117997 Moscow, Russia; amosovasg@yandex.ru

**Keywords:** fractality, mathematical model, sensors, navigation and motion control, telecommunication systems and networks, flaw detection, estimation, hurst parameter

## Abstract

In this paper, the fractal properties of stochastic processes and objects in different areas were specified and investigated. These included: measuring systems and sensors, navigation and motion controls, telecommunication systems and networks, and flaw detection technologies. Additional options that occur through the use of fractality were also indicated and exemplified for each application. Regarding the problems associated with navigation information processing, the following fractal nature processes were identified: errors of inertial sensors based on the microelectromechanical systems called MEMS, in particular gyroscopic drift and accelerometer bias, and; the trajectory movement of mobile objects. With regard to navigation problems specifically, the estimation problem statement and its solution are given by way of the Bayesian approach for processing fractal processes. The modified index of self-similarity for telecommunication series was proposed, and the self-similarity of network traffic based on the R/S method and wavelet analysis was identified. In failure detection, fractality manifested as porosity, wrinkles, surface fractures, and ultrasonic echo signals measured using non-destructive sensors used for rivet compound testing.

## 1. Introduction

Many processes in natural and artificial systems are known to have fractal structures [1,2]. Currently, the fractal properties of objects and processes continue to be studied in many areas [3]. With the development of higher-precision sensors and improved measuring and diagnostic systems, a need to improve our understanding of fractal structures and system errors has arisen. One goal of contemporary research in this area is to improve the cybersecurity of modern systems. The use of fractality allows mathematical models of processes and objects to be refined, thereby helping to overcome problems of analysis and synthesis to achieve greater competence. 

This article examines the features of stochastic process models with fractal properties and their applications in some important areas. These areas include: sensors and measuring systems [4,5], navigation and motion controls [6,7], telecommunication systems [3,8,9], and flaw detection technologies [10,11].

The structure of the paper is as follows: processes and objects of a fractal nature in key areas. The possibility of using fractal properties to improve the quality of problem solving is then by evaluated. Following this, the positive effects of fractality in these contexts is considered.

## 2. Fractal Process Model

First-order Markov processes have been widely used in practice. However, there are others random processes with short- and long-term memory.

One possible model by which to understand the higher-order Markov process as it applies to memory, is a model of fractional Brownian motion [12,13] (fractional Wiener process—FWP) containing the Hurst parameter.

To identify the features of the fractional structure of the process, fractality indicators such as the Hurst parameter are used. Several methods are used to estimate the Hurst parameter, including: rescaled range analysis (the R/S method), variance change graph analysis, and wavelet analysis [13,14]. According to the R/S method, the Hurst parameter of a time series is calculated by the following formula:

(1) $$H=\ln\left(\frac{R}{S}\right)/\ln\left(\frac{n}{2}\right),$$

where *R* is the range of the first *n* cumulative deviations from the mean, *S* is the standard deviation of observations, and *n* is the number of observations.

The FWP is called a random process x(t) with Hurst parameter *H* (0<H<1) if $\Delta x=x(t)-x(t_{0})$ has a Gaussian distribution with zero mathematical expectation and variance $\sigma^{2}\cdot {\left(t-t_{0}\right)}^{2H}$, where *σ* is a positive constant, namely its probability measure [1].

(2) $$P(\Delta x<X)=\frac{1}{\sqrt{2\pi }\sigma \cdot \;{\left(t-t_{0}\right)}^{H}}\cdot \int_{-\infty }^{X}\exp\left[-\frac{1}{2}{\left(\frac{u}{\sigma \cdot {\left(t-t_{0}\right)}^{H}}\right)}^{2}\right]du$$

at *H* = 0.5, the FWP is consistent with classical Brownian movement (the classical Wiener process—CWP). The increments $\Delta x=x(t)-x(t_{0})$ of fractional Brownian motion are called fractional Gaussian noise, with variance obeying the ratio $D[x(t)-x(t_{0})]=\sigma^{2}\cdot {\left(t-t_{0}\right)}^{2H}$.

At Hurst parameter *H*: (1) if 0<H<0.5, then the process has anti-persistent properties, when an upward trend is replaced by a downward trend or vice versa; (2) if H≈0.5, then the process is consistent with the classical Brownian movement; (3) if 0.5<H<1, then the process has persistent (i.e., trend-resistant) properties.

## 3. Mathematical Model of Inertial Sensor Errors in Navigation Systems with Respect to the Chaotic Indicator

Due to the development of high-performance navigation and motion control systems, the requirements for estimating the accuracy of true process parameters that determine the qualitative characteristics of these systems have increased. When processing navigation information parameters by way of an inertial navigation system, the requirements for precision features can be met by making system error descriptions precise [4,6]. 

When processing navigation information, it is important to take into account the characteristics of errors in the output signals of navigation system sensors. The Wiener process is widely used to describe modeling errors of inertial sensors with stochastic features [15]. In particular, such a model is used to describe the zero drift of a gyroscope.

The Wiener process model [16] for an error x(t) in continuous time is

(3) $$\dot{x}(t)=w(t),$$

where

(4) E[w(t)]=0,E[w(t)w(τ)]=q(t)δ(t−τ),

where q(t) is the intensity of continuous white noise w(t). 

The discrete-time state equation with a sampling period $T=t_{i}-t_{i-1}$

(5) $$x_{i}=x_{i-1}+w_{i}$$

The covariance Q for discrete noise $w_{i}$ in case q(t)=q=const

(6) $$Q=\sigma^{2}=qT.$$

The process $w_{i}$ can be represented by a model using white noise $n_{i}$ with unit intensity via ratio $w_{i}=\sqrt{qT}n_{i}$.

It should be noted that the process x(t) described by a stochastic differential Equation (3) is a Wiener process defined through the white noise w(t). A sequence $x_{i}$ (5) is a sequence with uncorrelated increments and is called a Wiener sequence.

Based on the results of other authors’ experimental analyses, error behavior graphs of inertial sensor outputs suggest that error description processes have a fractal nature.

This is indicated by the results of gyroscopic drift testing presented in [17]. Constant and variable components, correlation coefficients, variance within a drift variable, and white noise were taken into account in describing the gyroscopic drift model [17]. The paper [15] also proposes a mathematical model of sensor error based on Allan variation, which uses unknown parameters. The model characterizes the contribution of the error components of the sensor, corresponding to a linear trend, Wiener process, flicker noise, white noise and quantization noise.

Recently, interest in the class of non-stationary processes that have a fractal nature has deepened [1,2,3,5,6,7,8,9,10,14,18,19].

***Models of sensor errors based on fractal Wiener process***

Choosing a probability measure (2) for increments of a random process $\dot{x}(t)=w(t)$, $x_{i}=x_{i-1}+w_{i}$, $\Delta x=x(t)-x(t_{0})=x_{i}-x_{i-1}$, we obtain a model with the FWP for an error.

***Sensor Error Measurement Model***

Based on the obtained results of analyzing gyroscope and accelerometer tests, a new mathematical model of inertial sensor error based on the fractal Wiener process is proposed considering given the chaotic indicator

(7) $$y_{i}=\eta_{i}+x_{i}+v_{i},$$

where $\eta_{i}$ is the trend component, $x_{i}$ is the component expressing entropy of a process, modeled by the FWP in accordance with (2) and dependent on *H*—the Hurst parameter;$v_{i}$ is the random white noise with variance $R_{i}$.

### 3.1. Experimental Confirmation of Fractal Properties of Gyroscope Zero Drift Process

**Figure 1:**
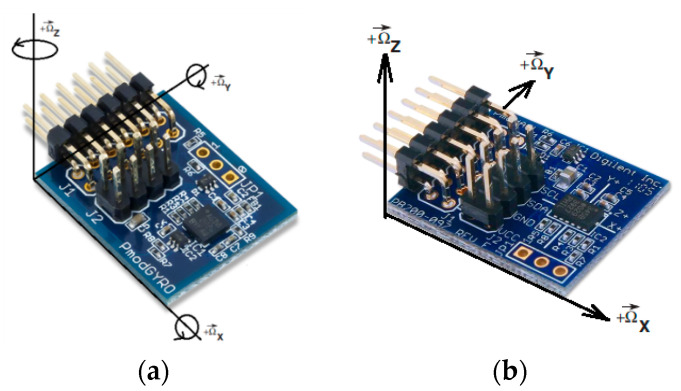
Inertial sensors: (**a**) gyroscope L3G4200D; (**b**) accelerometer ADXL345.

The digital gyroscope STMicroelectronics L3G4200D PmodGYRO (Figure 1a) was examined using a myRIO National Instruments (NI) device utilizing its onboard FPGA, a dual-core programmable controller, and LabVIEW software environment [20]. The L3G4200D is a low-power, three-axis, angular rate sensor able to provide unprecedented, zero bias stability (zero-rate level), and sensitivity stability over temperature and time [21]. 

Measured range: one of the available value ranges, e.g., ±250 dps, or °/s (grad/s). The sensitivity value is 0.00875°/LSB (LSB—least significant bit).

**Figure 2:**
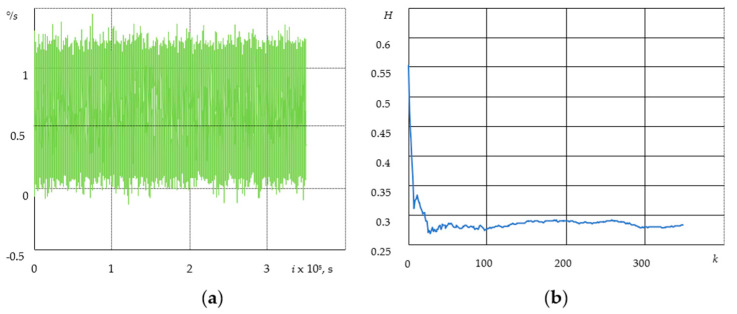
Gyroscope drift according to X coordinate (**a**) and the Hurst parameter with the gyroscope drift for 1 h (**b**).

Figure 2a shows the gyroscope drift at the following parameters: data transfer rate is 100 Hz; sampling time is 1 h with sample spacing $T=10^{-2}$ s—360,000 values at a sensitivity of 250 dps. The zero bias (digital zero-rate level) is more than 0.5 °/s.

In our experiment (Figure 2a), a signal is shown at the output of the electronic part of the sensor where the noise component (bias instability) is caused by electronic components that are part of an electrical circuit.

The value of the Hurst parameter *H* was calculated by the method based on wavelet analysis. We used the standard MatLab wfbmesti function in accordance with previous approaches [22,23,24]. As can be seen from Figure 2b, the value of the *H* parameter varies by about 0.28, i.e., the process has anti-persistent properties.

### 3.2. Modeling of Accelerometer Bias

The digital accelerometer ADXL345 [25] (Figure 1b) is examined using the myRIO NI device and the LabVIEW software environment. The accelerometer was placed on a fixed insulated base. The output signals were registered along one of the sensitivity axes, orthogonal to the gravitational vector g.

**Figure 3:**
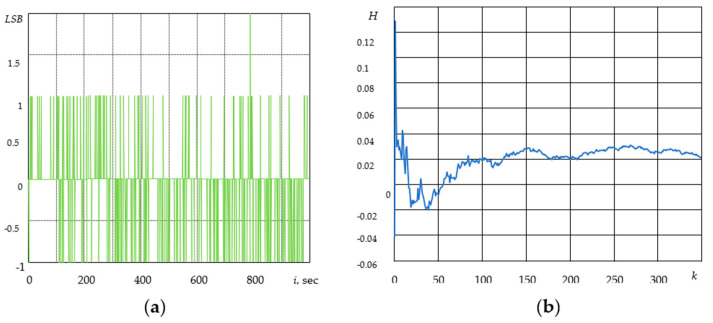
Accelerometer bias and drift by *x* coordinate (**a**), Hurst parameter (**b**).

Figure 3a shows the accelerometer bias at the following parameters: 16 g resolution range; 0.0625 g/LSB scale; 100 Hz data transmission rate; 20 min sampling time with $T=10^{-2}$ s—127,077 sample spacing values; and the temperature of 23 °C, LSB = 2 g/2^10^.

Figure 3a shows the signal at the output of the accelerometer, where the noise component of bias instability is in the range of −1 to +1 LSB, which is caused by the electronic part of the sensor [25].

When examining the accelerometer bias for 20 min (Figure 3b), the Hurst parameter fluctuates by about 0.03. Therefore, the process has anti-persistent properties.

In inertial sensors, changes in the Hurst parameter from small values to higher ones can serve as an indicator that errors of inertial systems accumulate due to the departure of the zero bias because of technical malfunctions, temperature influence, and vibration. For different types of inertial sensors (gyroscopes, accelerometers), the Hurst parameter will be different.

## 4. Navigation and Motion Control

The accuracy of parameter estimation in orientation tracking is mostly determined by the choice of mathematical, moving object, descriptions, and the choice of efficient algorithms for nonlinear filtering. In both cases, when solving the problem of trajectory tracking, the mathematical models based on the classical Wiener process are widely applicable. At the same time, an aggregation in the form of the FWP is used to model stochastic processes with fractal properties [1,18].

***Mathematical Models of Object Movement for Trajectory Tracking Problems***

The object moves in a horizontal plane with coordinates *x* and *y* of Cartesian axes. Let us consider the motion model for one coordinate *x*; the model for another coordinate, *y*, is similar. 

Let us also consider the object movement models traditionally used to solve orientation tracking problems [16,18,26,27].

Model with Wiener process for acceleration

(8) $$\dddot{x}=w(t),$$

with white noise characteristics w(t).

Model with white noise for acceleration, or Wiener process for speed

(9) $$\ddot{x}(t)=w(t).$$

A model with white noise for speed, or a Wiener process for a coordinate, is determined by Equations (3) and (4).

It is essential that all presented models of motion, (3), (8), and (9), are defined through the Wiener process, and that their discrete representations with a period of discretization $T=t_{i}-t_{i-1}$ in the form of $x_{i}=\Phi_{i}x_{i-1}+w_{i}$, are defined through the Wiener sequence (5). Here, $w_{i}$ is an *n*-dimensional-centered Gaussian white noise sequence with the covariance matrix $Q_{i}$. $\Phi_{i}$ is the known matrix of a respective dimension. 

With this in mind, we propose *new models of object motion* based on the use of the fractional Wiener process (2) [18,28].

***Models of object movement based on the FWP***

By choosing the probability measure (2) for increments of a random process

$$\dot{x}(t)=w(t),x_{i}=x_{i-1}+w_{i},\Delta x=x(t)-x(t_{0})=x_{i}-x_{i-1},$$

we obtain a model with the FWP for the coordinate. Proceeding similarly for processes (8) and (9), we obtain a model with the FWP for acceleration and a model with the FWP for speed, respectively.

***Measurement model for trajectory tracking***

A range and azimuth tracking approach can be used to determine the location of an object.

Measuring range ($\rho_{i}$) and bearing ($\alpha_{i}$)

$$\xi_{i}=\left[\begin{array}{c} \xi_{1i}\\ \xi_{2i} \end{array}\right]=\left[\begin{array}{c} \rho_{i}\\ \alpha_{i} \end{array}\right]=\left[\begin{array}{c} s_{1}(x_{i},y_{i})\\ s_{2}(x_{i},y_{i}) \end{array}\right]+\left[\begin{array}{c} \Delta \rho_{i}\\ \Delta \alpha_{i} \end{array}\right]=\left[\begin{array}{c} \sqrt{x_{i}^{2}+y_{i}^{2}}\\ arctg(y_{i}/x_{i}) \end{array}\right]+\left[\begin{array}{c} \Delta \rho_{i}\\ \Delta \alpha_{i} \end{array}\right].$$

To obtain a linear model of measurements, the primary measurements of the polar coordinates of an object, range ($\rho_{i}$), and bearing ($\alpha_{i}$), can be converted into a rectangular coordinate system and are presented in the following form [18,26]

(10) $$x_{i}^{*}=\underset{x_{i}}{\underbrace{\rho_{i}\cos\alpha_{i}}}+\Delta x_{i};\;y_{i}^{*}=\underset{y_{i}}{\underbrace{\rho_{i}\sin\alpha_{i}}}+\Delta x_{i},$$

where $\Delta x_{i}$ and $\Delta y_{i}$ are random errors of coordinate measurements with the following characteristics

$$E(\Delta x_{i})=E(\Delta y_{i})=0;\;\sigma_{\Delta x_{i}}^{2}=E(\Delta x_{i}^{2})=\sigma_{\Delta \rho_{i}}^{2}\cos^{2}\alpha_{i}+\sigma_{\Delta \alpha_{i}}^{2}\rho_{i}^{2}\sin^{2}\alpha_{i};\;\sigma_{\Delta y_{i}}^{2}=E(\Delta y_{i}^{2})=\sigma_{\Delta \rho_{i}}^{2}\sin^{2}\alpha_{i}+\sigma_{\Delta \alpha_{i}}^{2}\rho_{i}^{2}\cos^{2}\alpha_{i};\;E(\Delta x_{i}\Delta y_{i})=R_{\Delta x_{i}\Delta y_{i}}=(\sigma_{\Delta \rho_{i}}^{2}-\sigma_{\Delta \alpha_{i}}^{2}\rho_{i}^{2})\sin\alpha_{i}\cos\alpha_{i}$$

Linear measurements will be used for the Kalman Filter (KF). Let us now consider an example of fractal process filtering.

## 5. Fractal Process Filtering

To clearly identify the features of filtration in the fractal process, we intentionally choose the simplest model to express the FWP. It is necessary to evaluate the coordinate of a fractal Wiener process

(11) $$x_{i}=x_{i-1}+w_{i}$$

using linear measures of type $y_{i}=x_{i}+v_{i}$, where i=0, 1, … are the moments of time and $w_{i},v_{i}$ are the zero-mean Gaussian white noises independent of each other and of $x_{i}$, with variances $Q=\sigma_{w}^{2}$ and $R=\sigma_{v}^{2}$, respectively. Here, the values of the process $x_{0}$ at the initial represent the random zero-mean Gaussian variable with covariance at the $P_{x_{0}}=\sigma_{x_{0}}^{2}$.

Modeling parameters: initial state $m_{x_{0}}=0$, $\sigma_{x_{0}}=20$; $\sigma_{v}=10$, discretization period T=1 s. The FWP (11) was modeled using the wfbm function in the MatLab mathematical package, which is based on wavelet decomposition and simulates a fractal Wiener sequence with a given Hurst parameter.

Kalman filtering and a non-recurrent Bayesian algorithm (BA) of optimal linear filtering were used to obtain the required estimate [28].

It is essential that if, for the classical Wiener process, increments *W_i_* are independent, then the FWP does not have this property and is not a Markov process, except in cases where *H* = 0.5 when the FWP is consistent with the CWP. In such cases, KF only gives the optimal solution for this value of *H* parameter and is not optimal with other *H* parameter values.

In general, when 0<H<1, optimal estimations of non-recurrent BA and expected (analysis) covariance matrices for estimation error $e_{i}=x_{i}-{\tilde{x}}_{i}(y_{i})$, are determined by means of the following expressions [28,29]:

(12) $${\tilde{x}}_{i}={\overset{-}{x}}_{i}+P_{x_{i}y_{i}}{\left(P_{{}^{y_{i}y_{i}}}\right)}^{-1}[y_{i}-{\overset{-}{y}}_{i}],P_{e_{i}}=P_{x_{i}x_{i}}-P_{x_{i}y_{i}}P_{y_{i}y_{i}}^{-1}P_{y_{i}x_{i}}$$

where $P_{a_{i}b_{i}}$ are covariance matrices.

The selective (valid) r.m.s. of the estimation errors are presented, calculated as

(13) $$\tilde{\sigma }{\;}_{i}^{\eta }\approx \sqrt{\frac{1}{L}\sum_{j=1}^{L}{\left(e^{\eta }{\;}_{i}^{\left(j\right)}\right)}^{2}},e^{\eta }{}_{i}^{\left(j\right)}={\tilde{x}}_{i}^{\eta }{}_{}^{\left(j\right)}(y_{i}^{\left(j\right)})-x_{i}^{\left(j\right)},\eta =BA,KF,L=3000.$$

**Figure 4:**
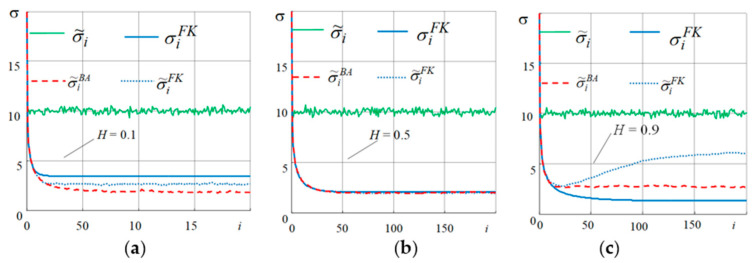
R.m.s. of estimation errors with different Hurst parameters (*H*.). (**a**) *H* = 0.1, (**b**) *H* = 0.5, (**c**) *H* = 0.9.

Modeling and estimation of the FWP process was carried out with different Hurst parameters: *H*: 0.1; 0.5; 0.9 (Figure 4). The number of measurements can be expressed as $i=\bar{1.200}$.

As Figure 4a–c show $\sigma_{i}^{FK}$ is the expected r.m.s of estimation errors corresponding to the error variance of optimal estimation, which is the only element of the analysis matrix of KF covariance errors. ${\tilde{\sigma }}_{i}^{FK}$ is the selective r.m.s of filtering errors when using KF; $\sigma_{i}^{BA}$ is the expected r.m.s. of filtering errors when using non-recurrent BA; ${\tilde{\sigma }}_{i}^{BA}$ is the selective r.m.s. of filtering errors for BA, and ${\tilde{\sigma }}_{i}$ is the selective r.m.s. of measurement errors.

The results show the coincidence of the expected $\sigma_{i}^{BA}$ (12) and the valid ${\tilde{\sigma }}_{i}^{BA}$ (13) accuracy characteristics of the presented optimal linear algorithm for non-recurrent estimation. When parameter *H* = 0.1, higher estimation accuracy can be achieved with the help of the optimal linear algorithm than with KF. When the parameter *H* = 0.5, both KF and the linear optimal algorithm estimate the random process with equal accuracy. When parameter *H* = 0.9, for KF, estimation error is increased in comparison to the optimal linear algorithm. A mismatch between the results of analysis and the actual (selective) characteristic for KF is observed at *H* = 0.1 and *H* = 0.9, respectively.

The study shows that a higher estimation accuracy can be achieved using non-recurrent algorithms, unlike KF.

In navigation and motion control, the values of the Hurst parameter for object motion models may change due to changes in the state of the environment (e.g., turbulence, weather conditions, natural phenomena, etc.).

For Kalman-type algorithms to be efficient, it is necessary to solve the adaptive problem of estimation by introducing and evaluating an unknown parameter—the fractal parameter *H*.

## 6. Information and Telecommunication System

The development and research of mathematical models of network telecommunication traffic allow us to define the boundaries of network performance and optimize the network structure of the system to minimize queueing delays. Traditional network traffic models show pulsations on short time scales but very smooth traffic at larger scales. Regardless, traffic shows variability over a wide range of time scales. When studying network telecommunication traffic, one of the important tasks is to identify anomalies and respond to them early.

Analyses of network telecommunication traffic result in time series (TS) analyses most widely used in traditional methods of statistically analyzing random variable functions. Alongside with traditional methods the signal processing methods based on fractal and wavelet-transformations [5,7,9,10,14,18,19], have seen more widespread use in recent years. The first of these analyses is specifically concerned with the identification of fractal properties that allow time series’ to be attributed to predetermined models. One distinct benefit of these analyses is their ability to reveal features of local structures and the different properties of complex signals that would otherwise be invisible using standard, real-time representations.

Telecommunication network traffic exhibits self-similar properties over a wide range of time scales that are very different to the properties of traditional models. Self-similar models are appropriate for telecommunication network traffic, as they have the capacity to estimate network performance/quality, allocate resources, and ensure quality of service [9].

Therefore, there is a need to develop new algorithms and modify those that exist for the purpose of analyzing the information systems (IS) of telecommunication series.

In order to analyze the telecommunication system, the use of an algorithm consisting of the following steps was proposed:

(1) Pre-processing of the traffic time series consisting of statistical data sampling with the purpose of generating the TS we are interested in;
(2) Estimation of fractality indicators using different methods such as R/S methods and wavelet analyses;
(3) Identification of the mathematical models of TS by approximation to known models, or through synthesizing the structures and parameters of mathematical TS models.

To reduce the possibility of misinterpreting results of the parameter *H* evaluation, we propose to analyze not only the entire sample, but also the individual blocks of the sample. In this context, we propose to consider the blocks separately with a window offset and/or in a progressive way. Depending on the parameter *H* estimation method, we will make further calculations for each concerned block(s) separately to determine the expected value of the results found. This modified Hurst parameter, in the author’s opinion, will more adequately reflect the real environmental issues associated with identifying the fractal properties of telecommunication time series.

The result of the analyses proposed here will provide full objective information about the telecommunication time series of IS, with the determined characteristics allowing for the identification of a mathematical TS model. To solve some practical problems, for example, the identification of anomalies in the network traffic that may impact IS data integrity, it is necessary to compare the TS with an identified model and compare the results.

**Figure 5:**
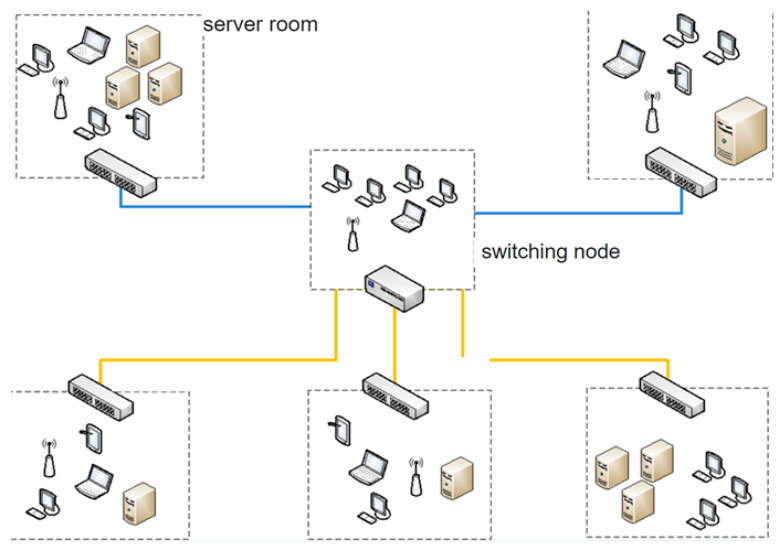
Scheme of the organization’s telecommunication system with incoming and outgoing Internet traffic.

In order to analyze telecommunication traffic, we will consider the time series of an organization’s incoming and outgoing internet traffic (Figure 5). The organization selected will have an average traffic volume of more than 160 Gbytes per day. 

Let us consider two time series of incoming and outgoing internet traffic for an organization with an average volume of more than 160 Gbyte a day. Let the first TS be collected over a sampling period of 60 days with sample spacing equal to 1 h, and let the second TS be sampled over 7 days, with sample spacing equal to 1 min. The parameter *H* can be obtained by charting the dependence of lg(E(R(n)/S(n))) on lg(n) and then using the obtained points to match a straight line with a slope of parameter *H* by the least squares method. Here, we will henceforth consider *E* the symbol of the expected value.

**Figure 6:**
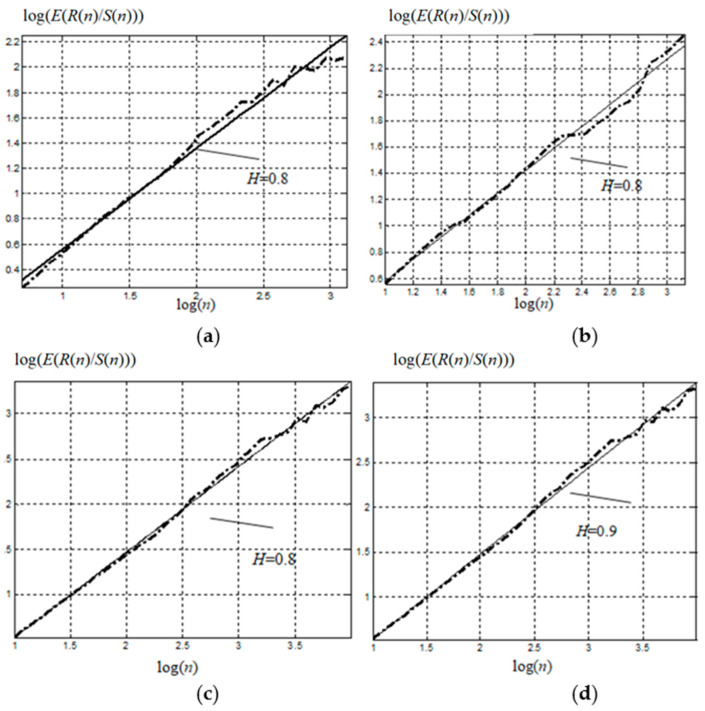
Characteristics of information and telecommunication systems: (**a**,**c**) show the analysis of 2-month, and weekly, series of intensity, respectively, in bytes; (**b**,**d**)—in packages.

Figure 6 shows the dependence of the standardized range logarithms on the logarithms of range lengths, and the linear regression with a slope of parameter *H* according to the *R*/*S*-method.

The time series were divided into blocks in a progressive way, with spacing of 100 discrete points, i.e., the first block included 100 values, the second included 200, the third included 300, etc. Each block, in turn, was divided into intervals ranging in size from 10 discrete points to a 20% block length increase in size between adjacent intervals. For each interval, the *R*/*S* ratio and the *R*/*S* mathematical expectation of the given block were calculated.

While investigating the information and telecommunication systems, the parameter *H* ≥ 0.8. Therefore, this process has persistent properties. Because of this, we propose to use fractality indicators for the time series model description, along with various traffic indicators (byte intensity per time unit, batches intensity per time unit, average batch size, throughput, loss rate, channel delay, loading, etc.).

In addition to the network traffic model, the fractality indicators can also be used to determine threats to information resources by solving a network classification problem [19]. Thus, the paper [19] proposes the modeling and implementation of a threat protection system with an attack forecast model mode of adaptation and parameter setting based on intelligent technologies in addition to fractal and wavelet analyses.

Thus, in telecommunication systems, the Hurst parameter can serve as an indicator when abnormal influences occur in the system (unauthorized access, computer attacks, etc.).

## 7. Defect Detection

Today, developments relating to the transition to advanced digital intelligent manufacturing technologies, robotic systems, big data processing systems using artificial intelligence, and machine learning continue to actively develop. In every industry, the use of non-destructive testing methods is able to investigate components’ properties on site or in the field. Non-destructive testing methods appeal because they are much cheaper and faster than traditional, destructive, tests and do not cause any damage to the constructions’ components.

Methods of image recognition using photo- and camcorder-sourced images are used to detect defects [30,31]. Another effective approach is an ultrasonic method of non-destructive testing [32,33], which can be operated using flaw detectors. 

Fractality manifests as porosity, wrinkles, and surface fractures, all of which can be detected during organoleptic testing and by ultrasonic echo signals by non-destructive control of rivet joints.

The analysis of non-destructive testing signals was carried out to identify their fractal properties.

**Figure 7:**
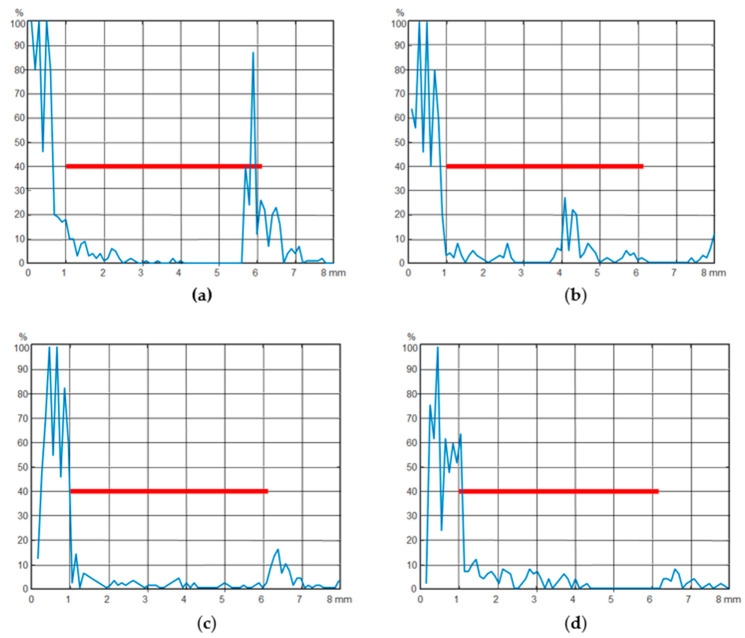
Echo signals of ultrasonic flaw detector during non-destructive testing of rivet joints: (**a**) is a normal rivet; (**b**) defect in the middle of the rivet at the 80% undercut; (**c**) defect in the middle of the rivet at the 50% undercut; (**d**) defect at the rivet cap base at the 50% undercut.

Figure 7 represents the observed echo signals during non-destructive testing using an ultrasonic flaw detector (Olympus EPOCH LTC) with an accuracy of measuring rivet length up to 0.01 mm. The accuracy of measuring the echo signal amplitude is up to 0.25%.

The red line is a strobe for detecting defects. Rivet defects differ in the type of echo signal in relation to this strobe.

The Hurst parameter was calculated for 28 implementations of echo signals of an ultrasonic flaw detector for each of the normal groups, and for three groups with defective rivet types. For normal rivet joints, the parameter *H* was approximately 0.2, whereas for all the defective rivets it was about 0.35. This can serve as a reliable indicator of the presence or absence of a defect in binary classification. As we have shown in [34] in the case of binary classification, 100% accuracy can be achieved using both deep neural networks with an LSTM layer, and by estimating the Hurst parameter *H* for the same source data.

## 8. Conclusions

Following the specification and investigation of the fractal properties of processes and objects in different areas, the present study showed that when constructing models of processes and objects in various domains, proper allowances must be made for fractality.

A stochastic modeling process based on the fractal Wiener process was proposed, with provision for the Hurst parameter.

Relevant to the problems of navigation information processing, the following fractal nature processes were identified: errors of inertial sensors based on the microelectromechanical systems called MEMS, in particular the gyroscopic drift and accelerometer biases, and the trajectory movement of mobile objects.

New mathematical models of inertial sensor errors based on the fractal Wiener process were presented.

New models of object motion based on the use of the fractional Wiener process were also proposed. The specific nature of estimating fractal process parameters using Kalman-type and non-recurrent Bayesian algorithms were investigated. Our illustrative examples show that, when estimating the state of the studied processes, fractality must be accounted for.

In addition, we revealed that the traffic in information systems has a fractal nature. A modified index of self-similarity for telecommunication series was proposed, and we identified network traffic self-similarity based on the R/S method and wavelet analysis. At the same time, we proposed to use the fractality indicator when identifying threats to information resource protection systems.

In failure detection, fractality is manifested as porosity, wrinkles, surface fractures, and ultrasonic echo signals through the non-destructive testing of rivet compounds. We recommend using the Hurst parameter when seeking the reliable binary classification of defects in rivet joints.

## Data Availability

https://www.scopus.com/authid/detail.uri?authorId=6505799329 (accessed on 2 September 2021).
